# The Influence of Protein Charge and Molecular Weight on the Affinity of Aptamers

**DOI:** 10.3390/ph16030457

**Published:** 2023-03-18

**Authors:** Alissa Drees, Tung Lam Trinh, Markus Fischer

**Affiliations:** 1 Hamburg School of Food Science, Institute of Food Chemistry, University of Hamburg, Grindelallee 117, 20146 Hamburg, Germany; 2 Center for Hybrid Nanostructures (CHyN), Department of Physics, University of Hamburg, Luruper Chaussee 149, 22761 Hamburg, Germany

**Keywords:** aptamer–protein interaction, ionic binding, isoelectric point, molecular weight, SELEX, aptamer database

## Abstract

Aptamers offer several advantages over antibodies. However, to ensure high affinity and specificity, a better understanding of the interactions between the nucleic-acid-based aptamers and their targets is mandatory. Therefore, we investigated the influence of two physical properties of proteins—molecular mass and charge—on the affinity of nucleic-acid-based aptamers. For this purpose, first, the affinity of two random oligonucleotides towards twelve proteins was determined. No binding was observed for proteins with a negative net charge towards the two oligonucleotides, while up to nanomolar affinity was determined for positively charged proteins with a high pI value. Second, a literature analysis comprising 369 aptamer–peptide/protein pairs was performed. The dataset included 296 different target peptides and proteins and is thus currently one of the largest databases for aptamers for proteins and peptides. The targets considered covered isoelectric points of 4.1–11.8 and a molecular weight range of 0.7–330 kDa, while the dissociation constants ranged from 50 fM to 29.5 µM. This also revealed a significant inverse correlation between the protein’s isoelectric point and the affinity of aptamers. In contrast, no trend was observed between the affinity and the molecular weight of the target protein with either approach.

## 1. Introduction

Aptamers are in vitro selected single-stranded nucleic acid oligomers that fold intramolecularly into three-dimensional scaffolds and can recognize and bind to a specific target of interest. While aptamers achieve similar affinity and specificity compared to antibodies, nucleic-acid-based aptamers offer several advantages over antibodies. These include automated chemical synthesis, reversible folding, distinct thermal stability, low manufacturing costs, and high batch stability due to adjustable chemical parameters [1,2].

The selection of nucleic-acid-based aptamers was first described in 1990 by Tuerk and Gold, by Robertson and Joyce, as well as by Ellington and Szostak in three separate publications [3,4,5]. It classically follows the Darwinian evolutionary principle consisting of sequence variation, selection, and replication and is therefore known as the “Systematic Evolution of Ligands by EXponential enrichment” (SELEX). Over the past three decades, many variants of SELEX have been established. However, common to all SELEX methods is that the selection itself remains a “black box”—largely random and incomprehensible. Recently introduced high-throughput selection methods are increasingly providing insight into the process [6,7]. In addition, the first systematic studies and database analyses were conducted regarding the influence of selection conditions on the selection process [8]. Nevertheless, there is still no comprehensive understanding of the influence of selection parameters and interaction partners’ properties on the affinity and specificity of the selected aptamers. While aptamers have been selected for targets ranging in size from ions to whole cells, proteins are the most common type of targets [9,10,11]. Therefore, in the present study, we focused on the influence of two properties of peptides or proteins—molecular mass and pI value/charge—on the interactions with aptamers.

Aptamers have been shown to interact with their cognate target through a combination of hydrogen bonds, hydrophobic interactions, π-stacking, and electrostatic interactions [12]. The latter, in particular, are presumably dependent on the charge of the target. Therefore, a positive correlation between the charge of the target and the strength of the bond can be hypothesized. Indeed, previously, an inverse correlation between the protein charge and aptamer *K*_D_ was observed by Ahmad et al. [13], as the selection of aptamers for the protein PDGF-BB at different pH values of the selection buffer using the otherwise same selection protocol resulted in different affinities of the selected aptamers. McKeague et al. [8] also described an inverse correlation between the affinity of aptamers and the pH value of the selection buffer following a database analysis comprising, inter alia, 81 different target peptides and proteins. This implies that aptamers may have improved affinity to their target when applied in a buffer with a higher pH value. However, besides the net charge of the protein, the pH value can influence physicochemical properties such as intrinsic stability and the structure of both the nucleic acids and targets. It has been demonstrated for several targets that these can also significantly alter the protein–aptamer interaction [14,15,16,17,18]. Therefore, the use of the pI value at a constant pH is presumably a more suitable measure to assess the influence of the targets’ charge than the pH value. Contrary to the expectation, in a literature review of 75 aptamer–protein pairs, Ahmad et al. [13] found no trend between the isoelectric point of proteins and the affinity of aptamers.

Besides the charge, according to the shape space model, a greater number of distinct binding sites may lead to a larger share of potential high-affinity binders in a randomized pool and thus a greater chance of selecting such high-affinity binders [19,20]. It is therefore reasonable to hypothesize that the affinity of aptamers increases with the size of the target. Such a trend has so far only been described for aptamers for small molecules (<400 Da) by Carothers et al. [21], and to the best of our knowledge not been analyzed within other target types.

## 2. Results and Discussion

To analyze the influence of the charge and the molecular weight of proteins on the affinity for aptamers, first, the interactions of two oligonucleotides with randomly generated sequences (50 nt and 70 nt) with twelve proteins were screened using electrophoretic mobility shift assays (EMSA) and binding was confirmed and quantified using bead-based affinity chromatography in conjunction with a fluorescence assay. The proteins studied (cf. Table 1) were chosen to cover a broad range of the analyzed parameters while being easily available in high concentration. Precisely, they covered molecular weights in a range of 14.3–69.3 kDa and isoelectric points of 3.87–11.4, while the binding assays were performed at pH 7.4. Only the proteins lysozyme C, chymotrypsin (CTRC), and cytochrome c (CytC) showed significant binding, as indicated by the absence of the band of unbound aptamers (see OSF repository, cf. Data Availability Statement). Thus, no interaction was detectable when the protein had a negative net charge, whereas binding was identified when the proteins’ net charge was positive. As summarized in Table 1, the observed trend of binding of the 12 investigated proteins is in full concordance with the net charge of the proteins during the assays, further indicating a positive correlation between affinity and the isoelectric point of the proteins. In comparison, the molecular weight of the protein did not seem to have significant effect on the observed interactions. Noteworthily, the affinity of the oligos for lysozyme was calculated in the low nanomolar range, which was not expected, since SELEX-selected aptamers have already been reported whose binding was determined to be in a similar order of magnitude [22,23]. However, the direct comparability of the calculated *K*_D_ values across different affinity determination methods is most likely not guaranteed, and since for the present study only the resulting trend of the *K*_D_ value was of interest, we refrained from a comparison of the affinities with published aptamers [24].

**Figure 1:**
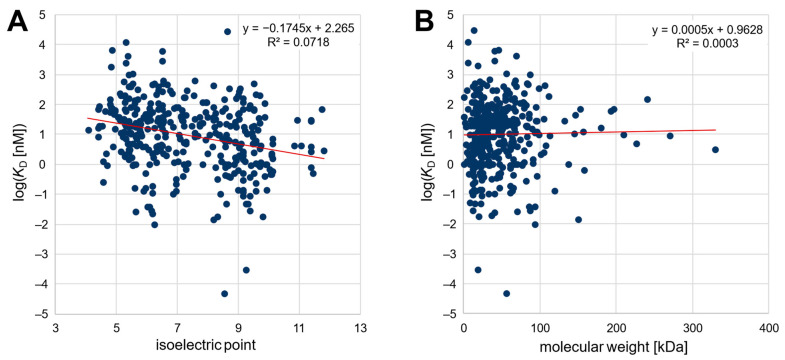
(**A**) Plot of the affinity of 369 aptamers against the isoelectric point and (**B**) against the molecular weight of the target peptide/protein.

Second, an extensive analysis of published SELEX data was carried out to determine a possible correlation between the affinity of various published nucleic-acid-based aptamers and the isoelectric point as well as the molecular weight of the target protein or peptide. For this purpose, we tried to analyze published aptamers with given affinity to their peptide/protein targets as comprehensively as possible. Thereby, the best published aptamer candidates from 369 distinct SELEX procedures were considered. The dataset comprised 296 different target peptides and proteins and is thus currently one of the largest databases for aptamers for proteins and peptides (openly available in the OSF repository, cf. Data Availability Statement as well as in the Supplementary Material). The targets ranged from a theoretical pI of 4.4 to 11.8 and covered molecular weights of 0.7–330 kDa, while the dissociation constants ranged from 50 fM to 29.5 µM. As shown in Figure 1, the published affinity of the aptamers was plotted logarithmically against the pI value and the molecular weight of the target protein. The relation between affinity and pI could be described by a linear regression function with a significant negative slope (−0.1745, *p* < 0.05). This confirms the initial hypothesis of a negative correlation between the isoelectric point of the target protein and the affinity (given as *K*_D_ value) of nucleic-acid-based aptamers. In contrast, as also observed in the section above, there was no correlation (*p* < 0.05) between the affinity of aptamers and the molecular weight of their target peptide/protein.

The literature review is biased to some extent because most often only successful selections that generate high-affinity aptamers are published. In addition, the affinity reported as the *K*_D_ value is not only highly dependent on the selection conditions but can also be significantly affected by the method chosen to determine the affinity, making the comparability of published values uncertain [24]. Nonetheless, because the variance is independent of the targets’ characteristics, the trend observed for the 369 aptamer/protein pairs should be valid despite the low coefficient of determination (R^2^ = 0.0718). In addition, it should be noted that the pI values in the literature review cannot be directly related to the charge, since the pH values during the assay of the affinity determination of the respective publication were not considered (see Figure 1A). However, it was verified that the pH value was in a similar range for most of the publications (typically pH 6.8–8.0), so it should be possible to infer the observed correlation of the pI value from the charge on the target. Noteworthily, none of the 296 target proteins/peptides for whom successful aptamer selections have been described have a theoretical pI lower than 4.4, although several proteins with a lower pI value exist, possibly revealing the difficulty of selecting unmodified high-affinity aptamers for proteins with a high negative net charge due to ionic repulsion.

## 3. Materials and Methods

If not specified otherwise, the chemicals were acquired from Carl Roth GmbH + Co. KG (Karlsruhe, Germany) or Sigma Aldrich Corp. (St. Louis, MO, USA). Agarose was acquired from GENEON GmbH (Ludwigshafen, Germany). Magnetic beads were acquired from chemicell GmbH (SiMAG-Carboxyl, 1.0 µM; Berlin, Germany). Lysozyme C (hen egg white), cytochrome c (equine heart), chymotrypsin (bovine pancreas), trypsin (porcine pancreas), ovalbumin (hen egg white), casein (bovine milk), pepsin (porcine gastric mucosa), lectin A (*Pseudomonas aeruginosa*, PA-I), and myoglobin (equine heart) were acquired from Sigma Aldrich Corp. (St. Louis, MO, USA). BSA was acquired from New England Biolabs Inc. (Ipswich, MA, USA). The lumazine protein of *Photobacterium leignathi* was expressed as described by Illarionov et al. [27]. A recombinant non-toxic double-mutant of *Pseudomonas* Exotoxin A (PEA; R276G and E553D) with a His-Tag was expressed in *Escherichia coli* BL21 (DE3) cells and purified via IMAC. This was conducted according to the protein expression protocol described by Frohnmeyer et al. [28] but with a pET-22b(+)-vector containing the gene coding for the recombinant PEA, which was synthesized by GenScript Biotech Corp. (Piscataway Township, NJ, USA). The sequences of oligonucleotide 1 (5′ATAAATTTAAGACATGAAAAAATAAATTTTTATTTTTTTACGTTTTTATT3′) and 2 (5′CGGTCCTCAGATGTGATTCCATCCTTCTTTTGAGCAAACTACCTGTA TAACGTAAGTCCGTGTGTGCGAA3′) were randomly generated and synthesized by Integrated DNA Technologies, Inc. (Coralville, IA, USA).

### 3.1. Electrophoretic Mobility Shift Assay

The EMSA was performed as described previously by Seo et al. [29] with minor modifications briefly described as follows. First, for screening, 500 nM of unlabeled oligonucleotide was combined with 200 µM of protein in a total volume of 20 µL per sample and incubated under shaking (440 rpm) for one hour at room temperature. For more detailed binding analyses for lysozyme C, chymotrypsin, and cytochrome C, the protein concentration in the samples was varied from 0–200 µM with at least 8 dilutions, while keeping the concentration of oligonucleotide constant at 500 nM (data not shown, since in the titration regime [24]). For all possible oligonucleotide–protein pairs, samples were prepared in triplicate in PBS (pH 7.4). EMSA was performed with a 5% (*w*/*v*) agarose gel where each sample was applied as a duplicate. For quantitative measurements, in addition to the samples, a calibration series of the corresponding oligonucleotide without protein (0 nM, 100 nM, 200 nM, 300 nM, 400 nM, 500 nM) was applied in duplicate in each lane of the gel. Electrophoresis was performed at 50 V for 30 min at room temperature in gel electrophoresis systems from Galileo Bioscience (Cambridge, MA, USA). Afterwards, the gels were stained overnight using GelRed. Visualization was carried out using a photo-documentation unit (Dark Hood DH-40/50, Biostep GmbH, Jahnsdorf, Germany) at a wavelength of 306 nm. Subsequently the share of unbound aptamers was determined using ImageJ (https://imagej.nih.gov/ij/index.html, last accessed on 10 January 2023).

### 3.2. Determination of Dissociation Constants via Fluorescence Assay after Bead-Based Affinity Chromatography

To avoid the titration regime, the *K*_D_ values were not determined via EMSA but using affinity chromatography, as described previously by Fischer et al. [24,30]. Briefly, the target protein was immobilized on the surface of carboxylated magnetic particles according to the manufacturer’s 2-step protocol. Fluorescent Alexa488-labeled oligonucleotides were diluted in a total volume of 90 µL with PBS to 1 nM, 5 nM, 10 nM, 20 nM, 40 nM, and 60 nM for lysozyme C and to 100 nM, 600 nM, 1 µM, 5 µM, 10 µM, and 20 µM for chymotrypsin and cytochrome c. Afterwards, 10 µL of target beads (10 mg/mL) were added to each dilution and the resulting solution was incubated for 60 min at room temperature under mild shaking and light exclusion. The supernatant was removed, and the beads were resuspended in 100 μL of double distilled water (ddH_2_O) and transferred into a microtiter plate for fluorescence measurement (SpectraMax2; extinction 485 nm, emission: 525 nm, Molecular Devices, LLC., San Jose, CA, USA). Every experiment was performed in triplicate and blank measurements were included. Data were fitted non-linearly (Hill-fit) using OriginPro 2019 software (OriginLab Corp., Northampton, MA, USA).

### 3.3. Statistical Analysis

Statistical analyses of the created aptamer database were performed using OriginPro 2019 (OriginLab Corp., Northampton, MA, USA) and Microsoft Excel 2019 software (Microsoft Corp., Redmont, WA, USA). Following linear regression, the regressions’ significance was determined based on their *p*-value as well as on the F-value of a performed analysis of variance (ANOVA).

## 4. Conclusions

Both the literature review and the experimental results ascertain the initial experimental observations of Ahmad et al. [13] and indicate that the charge of the protein has significant effect on the affinity of nucleic-acid-based aptamers. This can presumably be explained by the fact that the charge of the target determines the ionic interactions upon binding to negatively charged DNA aptamers. In addition, ionic bonds and ionic repulsion are stronger than most other bonds that describe the interactions of aptamers with their target [31]. This implies that it is more difficult for proteins with a low isoelectric point and consequently a negative net charge to select high-affinity aptamers than for proteins with a higher isoelectric point. Therefore, the net charge of the target proteins should be taken into account when evaluating the quality of an aptamer selection, e.g., by comparing the affinity of selected aptamers to the bulk *K*_D_ value of the initial library. Based on the correlation shown, it may also be possible to increase the affinity of aptamer pools by enhancing the ionic binding fraction with base modifications tailored to the charge of the protein [32].

On the other hand, no influence of the molecular weight of peptides and proteins on the affinity of aptamers was observed. This indicates that the generation of new—chemically similar, but structurally distinct—binding sites seems to have negligible influence on the number of suitable nucleic-acid-based binding species for the target.

## Figures and Tables

**Table 1:** Affinity of twelve proteins to two random oligonucleotides. N.d.: no binding determinable.

pI *	MW [kDa] *	Protein	Oligonucleotide	*K*_D_
11.4	14.3	Lysozyme C	1 2	14 nM 8 nM
9.59	53.7	Cytochrome C	1 2	1.4 µM 1.1 µM
8.75	25.7	Chymotrypsin	1 2	1.4 µM 3.3 µM
7.36	17.6	Myoglobin	1 2	n.d. n.d.
4.6–6.5	25.8	Trypsin-1	1 2	n.d. n.d.
5.82	69.3	BSA	1 2	n.d. n.d.
4.1–5.8	19–25	Casein	1 2	n.d. n.d.
5.36	68.8	PEA	1 2	n.d. n.d.
5.35	42.7	Ovalbumin	1 2	n.d. n.d.
4.94	12.8	Lectin A	1 2	n.d. n.d.
4.44	20.0	Lumazine Protein	1 2	n.d. n.d.
3.87	36.0	Pepsin	1 2	n.d. n.d.

* determined based on the sequences provided in the UniProt Knowledgebase [25] using the ProtParam tool (Expasy webserver [26]), if not provided by the manufacturer.

## Data Availability

The data included in the literature analysis as well as images of the EMSA and binding curves for lysozyme, chymotrypsin, and cytochrome c with the two oligonucleotides determined via affinity chromatography are openly available in the Open Science Framework repository (https://osf.io/7xznv/?view_only=30622a20dd264def98ff99674c89bc32, last accessed on 28 February 2023).

## Supplementary Materials

The following supporting information can be downloaded at: https://www.mdpi.com/article/10.3390/ph16030457/s1, Dataset S1: Dataset of the literature analysis comprising 369 aptamer–protein/peptide pairs.
